# ESPO and Academy for Gerontology in Higher Education Section Symposium: A New Normal in Teaching? Incorporating Unconventional and Creative Ideas Into Gerontology Curriculum

**DOI:** 10.1093/geroni/igab046.737

**Published:** 2021-12-17

**Authors:** Lauren Bouchard, Yan-Jhu Su, Marilyn Gugliucci

## Abstract

This symposium is intended to highlight novel, applied examples and classroom activities in gerontology curriculum. In accordance with the AGHE gerontological education competencies, these authors will provide insightful and fun connections to arts/humanities, popular culture, technology, and current events to inspire conversation, interest, self-reflection, and empathy in the classroom. The first author will discuss social media (e.g., TikTok) as a segue to difficult classroom conversations regarding negative stereotypes and ageism in society. Presenter two will discuss cross-field educational connections between music education and gerontology. Next, presenter three will put present a unique activity regarding technology, homeownership, and retirement with a competitive flair. Presenter four utilizes documentary to encourage empathy in nursing. Finally, presenter five will present a timely class debate regarding United States political office and ageism that is sure to create lively and relevant conversation.

